# Vaccine Literacy, COVID-19 Vaccine-Related Concerns, and Intention to Recommend COVID-19 Vaccines of Healthcare Workers in a Pediatric and Maternity Hospital: A Cross-Sectional Study

**DOI:** 10.3390/vaccines10091482

**Published:** 2022-09-06

**Authors:** Wakana Maki, Kazue Ishitsuka, Koushi Yamaguchi, Naho Morisaki

**Affiliations:** 1 Department of Social Medicine, National Center for Child Health and Development, Tokyo 157-8535, Japan; 2 Department of Immunology and Microbiology, Center of Maternal-Fetal, Neonatal and Reproductive Medicine, National Center for Child Health and Development, Tokyo 157-8535, Japan

**Keywords:** COVID-19 vaccine, vaccine literacy, healthcare workers

## Abstract

Vaccine literacy of healthcare workers (HCWs) may affect the COVID-19 vaccine uptake of the general population. This study aimed to clarify the vaccine literacy level of HCWs in Japan and its impacts on their concerns about vaccines and intention to recommend that others receive vaccines. This cross-sectional survey was conducted in July 2021 based on the recruitment of HCWs in a pediatric and maternity hospital and research center in Tokyo, Japan. All HCWs in this center had the chance to receive the COVID-19 vaccine before the survey, and their vaccine coverage was relatively high, at 95%. A total of 1519 workers answered the questionnaire. The results showed that HCWs with lower functional vaccine literacy had 1.5 times as many concerns about the efficacy of vaccines and 1.6 times as many concerns about the future side effects compared with those with higher literacy. Further, HCWs with higher vaccine literacy were more likely to recommend that older people, people with comorbidities, and pregnant women receive vaccines. Our findings suggest that high vaccine literacy alleviates concerns about COVID-19 vaccines and raised the intention to recommend vaccines to others. To achieve high vaccine coverage, countermeasures such as personalized education are essential.

## 1. Introduction

High uptake of COVID-19 vaccines is considered essential to end the COVID-19 pandemic. Inventing new vaccines is challenging and achieving high vaccine coverage is challenging and essential as well [1]. Beginning in late 2020, several vaccines were authorized, and vaccination programs started in many countries. Similarly, in Japan, on 17 February 2021, provision of vaccination started first among healthcare workers (HCWs) using the BNT162b2 mRNA (Pfizer/BioNTech) vaccine.

In light of the COVID-19 pandemic, COVID-19 vaccine acceptance of HCWs has gained attention, as current statistics show that vaccine acceptance of the general population correlates with that of the HCWs, and HCWs play a large role in achieving high vaccine coverage [2]. For instance, in Libya, 80% of HCWs answered that they would receive COVID-19 vaccines if the vaccine efficacy was 95%, and 86% answered that they would recommend that parents receive vaccines if the efficacy was high enough [3]. In Thailand, 96% of physicians had the intention to receive vaccines, and about 80% encouraged their family to receive the vaccine [4]. Thus, HCWs who were willing to receive COVID-19 vaccines did not always recommend that others receive the vaccines. However, the reasons for these gaps between the intentions to get the vaccine themselves and to recommend others to get the vaccine are not clear.

Whether people accept vaccination is related to vaccine literacy, which involves “not simply knowledge about vaccines, but also developing a system with decreased complexity to communicate and offer vaccines as sine qua non of a functioning health system” [5]. One previous study showed that people with high vaccine literacy were more willing to get vaccinated even after a major vaccine scandal [6], suggesting that elevating vaccine literacy of the population may improve the uptake of effective vaccines. However, the mechanism through which vaccine literacy contributes to high personal vaccine acceptance is not well known. Furthermore, the extent to which people with high vaccine literacy affect the surrounding people’s vaccine acceptance is also unclear. Therefore, this study focused on vaccine literacy of HCWs who have the possibility to influence the vaccine acceptance of the general population.

Accordingly, this study aimed to assess the vaccine literacy levels of HCWs in Japan, understand their impacts on the HCWs’ concerns before/after taking COVID-19 vaccines, and determine whether they would recommend that others (children, pregnant women, older people, people with comorbidities, and other adults) also receive vaccines. Achievement of these aims can help identify countermeasures to enhance the COVID-19 vaccine uptake; these results can help identify countermeasures to enhance the vaccine uptake of other vaccines as well.

## 2. Materials and Methods

### 2.1. Study Design and Participants

This cross-sectional survey was conducted during 13–20 July 2021, based on the recruitment of HCWs at the National Center for Child Health and Development in Tokyo, Japan. This national center consists of a pediatric and maternity hospital and a research center. The workers were recruited when they undertook regular health checkups in this center. The questionnaires were prepared and distributed to all workers who came for their regular health checkup during the predetermined period. No financial incentives were given. However, at the same time of this survey, respondents were able to get COVID-19 antibody tests and receive the results afterwards. Therefore, people who wanted to know the results of their COVID-19 antibody tests might have considered this an incentive to answer the questionnaire. Eligibility for this study was limited to HCWs who received regular health checkups at the National Center for Child Health and Development. This checkup was targeted toward all paid workers in this center. Therefore, guest research associates who mainly belonged to another hospital or research center were excluded. As the vaccine coverage in this survey was 95.3%, we limited our analysis to people who had received at least one COVID-19 vaccination dose. We felt that we lacked the power to observe the association among those who chose not to get vaccinated.

At the time of this survey in July 2021, most HCWs in Japan had access to vaccination, but most non-HCWs, that is, the general population, did not have access. In the center where this survey was conducted, all eligible people had the chance to receive the first and the second COVID-19 vaccine doses for free before this survey. In March 2021, medical doctors, nurses, and other HCWs who were at high risk for COVID-19 infection in the performance of their duties were given first priority for vaccination. Subsequently, other HCWs in this center had the chance to receive the vaccine before answering the questionnaire. On the other hand, at the time of this survey, most members of the general population, except for HCWs, such as older people (aged over 65 years) and people with comorbidities, had to wait for the opportunity to receive vaccines. Besides, at the time of this survey, only children aged 12 years or older were able to receive the BNT162b2 mRNA (Pfizer/BioNTech) vaccine, while only people aged 18 years or older were able to receive either the BNT162b2 or mRNA-1273 (Moderna) vaccine. In addition, vaccination of pregnant women and children was not prioritized in Japan at that time.

All procedures were conducted following the ethical standards of the Helsinki Declaration of 1975, as revised in 2013. Ethical approval was given by the Research Ethics Committee of the National Center for Child Health and Development, which reviewed the study protocol. An informed consent form was provided to and signed by the respondents before proceeding to the paper questionnaire.

### 2.2. Measurements

The self-reported questionnaire covered the following: demographic questions on sex, age, occupation, and whether the respondents were engaged in COVID-19 related jobs; the Kessler Psychological Distress Scale (K6) questionnaire [7,8], a validated short screening scale for non-specific psychological distress, questions about influenza vaccines, and questions about COVID-19 vaccines (see Table S1 for details).

#### 2.2.1. Vaccine Literacy

Table S1 presents the questionnaire for COVID-19 vaccine literacy. This questionnaire was built based on the Ishikawa test for chronic non-communicable diseases, which has already been validated [9]. This vaccine literacy scale was also used in online surveys in Italy [10] and Croatia [11] to assess COVID-19 vaccine literacy. Four items of the questionnaire aimed at assessing the respondents’ functional vaccine literacy skills, and eight items evaluated their interactive-critical vaccine literacy. Functional vaccine literacy refers to basic reading and writing skills [12] and leads to “improved knowledge of health risks and health services, and compliance with prescribed actions” [12]. Interactive-critical vaccine literacy refers to more advanced skills for problem-solving and decision-making [10,11].

Each response was given on a four-point Likert scale (1: never, 2: sometimes, 3: rarely, and 4: often) and scored using reversed items (1: often to 4: never). The score was obtained from the mean value of the answers to each scale (range: 1 to 4), with a higher value corresponding to a higher vaccine literacy level. In this survey, a Japanese-translated questionnaire was used. Answers to the questions about functional and interactive/critical vaccine literacy skills showed acceptable consistency (Cronbach’s alphas = 0.91 and 0.89, respectively).

Principal component analysis (PCA) was utilized to investigate how the questions on the functional and interactive-critical vaccine literacy scales were related to each other, and to assess whether the underlying components (factors) and each question’s load on the components could be identified as anticipated (as presented in Table S2). PCA showed two components accounting for 92.8% of the total variability. To clarify the relationships among the items, a varimax-rotation was applied, showing that all functional vaccine literacy questions loaded on one component, while all interactive-critical questions were loaded on the other component. A close correlation was observed between the questions within the functional scale and those within the interactive-critical scale.

#### 2.2.2. Vaccine-Related Questions

We also asked respondents whether they were concerned before taking COVID-19 vaccines when they decided to receive the dose or not, and whether they were concerned about vaccines at the time of our survey after taking the COVID-19 vaccine (see Table S1 for details). We defined concerns at these time points as pre-vaccination concerns and post-vaccination concerns, respectively. We also asked respondents whether they would recommend COVID-19 vaccines to others (children aged 18 years and under, older people, people with commodities, pregnant women, and other adults) on a four-point Likert scale (see Table S1 for details).

In the questionnaire about pre-vaccination concerns (see Table S1 for details), the respondents answered each question on a four-point Likert scale (always, sometimes, almost never, and never). We defined the people who answered “always” and “sometimes” as having pre-vaccination concerns, and people who answered “almost never” and “never” were defined as not having concerns. In the questionnaire about post-vaccination concerns (see Table S1 for details), the respondents answered with yes/no on whether they agreed with each question. We defined people who answered “yes” to each question as having post- vaccination concerns. In the questionnaire about intentions to recommend the COVID-19 vaccines to others (see Table S1 for details), the respondents answered each question on a four-point Likert scale (always, sometimes, almost never, and never). We defined respondents who answered “always” and “sometimes” to these questions as having intention to recommend vaccines to others, and people who answered “almost never” and “never” as not having such intention.

#### 2.2.3. Other Questions

We classified each demographic information into categories. Age was categorized into three groups: (i) less than 30 years, (ii) 31–50 years, and (iii) 51 years and older. Occupation was classified into five groups: (i) doctors, (ii) nurses, (iii) other paramedics, (iv) researchers and research assistants, and (v) office workers and others. In addition, respondents also answered the K6 questionnaire, which consists of six questions on a four-point Likert scale, with higher scores meaning greater depressed levels. The scores were categorized into four groups: (i) normal, 0–4, (ii) mild, 5–8 (iii) moderate, 9–12, and (iv) severe, 13–24 [7,8].

### 2.3. Statistical Analysis

All analyses were performed using Stata software version 16.0 (StataCorp LP, College Station, TX, USA).

We divided the functional and interactive/critical vaccine literacy scores into three groups: low, middle, and high literacy level using the tertiles of each score. Poisson multivariable regression models were used to examine the association between the outcomes and vaccine literacy levels, adjusting for gender, occupation, age, and K6 scores. For each analysis, two-sided *p*-values = 0.05 were considered statistically significant.

## 3. Results

A total of 1519 completed questionnaires were collected from 13–20 July 2021. As 1664 HCWs had the chance to participate in this survey, the response rate was 91.3%.

The main demographic characteristics of the respondents are shown in Table 1. Of the respondents, 76% were women. The respondents were aged 19–76 years, and their mean age was 38.0 years. About 26% were doctors, and 42% were nurses. Of the respondents, 24% showed symptoms of distress in the K6 screenings.

The mean score of functional vaccine literacy was 2.65 ± 0.73, and the interactive-critical score was 2.96 ± 0.62 out of a maximum of 4.

HCWs in the high vaccine literacy group were less likely to have vaccine-related concerns. For example, 27% of the HCWs in the low functional vaccine literacy group were concerned about serious life-threatening side effects, while 13% in the high vaccine literacy group did so. Similarly, HCWs in the high vaccine literacy group were more likely to recommend that others receive vaccination. For instance, 92% of the HCWs in the low interactive/critical vaccine literacy group answered that they would recommend COVID-19 vaccines to older people or people with commodities, while 97% in the high vaccine literacy group did so (see in Table 2 for details).

Table 2 shows pre-COVID-19 vaccination concerns for each vaccine literacy (functional and interactive-critical) level group. Low functional vaccine literacy was significantly associated with an increased risk of pre-vaccination concerns. On the contrary, there were no significant differences among different levels of the interactive-critical vaccine literacy group in pre-vaccination concerns.

Table 2 also shows the associations between vaccine literacy and post-vaccination concerns. HCWs with low functional vaccine literacy skills were significantly more concerned after vaccination that the vaccines might be ineffective compared to those with high vaccine literacy (risk ratio [RR], 1.54, *p* < 0.05). In addition, people with low functional vaccine literacy skills were significantly more concerned that the vaccines might do some harm in the future compared with people with middle and high vaccine literacy (RR, 1.62, 1.33, *p* < 0.05, respectively). Additionally, people with high functional vaccine literacy were significantly more willing to answer that they were not concerned about anything. People with middle and low functional vaccine literacy were significantly more likely to be concerned about side effects and serious subsequent complications. On the contrary, no significant relationships were observed between interactive-critical vaccine literacy skills and post-vaccination concerns, except for concerns about future harm among the high and low literacy groups.

As shown in Table 2, people with low functional vaccine literacy were significantly more unwilling to recommend COVID-19 vaccines to pregnant women and other adults than did people with high functional vaccine literacy (RR, 0.90, 0.98, *p* < 0.05, respectively). Additionally, people with low interactive-critical vaccine literacy were significantly more unwilling to recommend COVID-19 vaccines to older people, people with comorbidities, and pregnant women than did people with high literacy (RR, 0.97, 0.88, *p* < 0.05).

We also conducted continuous analysis in Table 3. Similarly, in the categorical analysis, higher vaccine literacy, particularly functional vaccine literacy, was mostly associated with decreased risk of pre-vaccination and post-vaccination concerns. However, regarding life-threatening side effects, people with higher interactive-critical vaccine literacy were significantly more concerned (RR, 1.19, *p* < 0.05). In addition, in terms of intention to recommend COVID-19 vaccines to others, higher vaccine literacy was associated with higher intention to recommend that others receive vaccination. Particularly, people with higher functional vaccine literacy skills recommended vaccines to pregnant women and healthy young people significantly more often. People with higher interactive-critical vaccine literacy recommended vaccines to older people, people with comorbidities, and pregnant women significantly more often. These results are consistent with the main categorical analysis.

## 4. Discussion

We found that low vaccine literacy in HCWs is associated with increased risk perception of low efficacy and serious adverse effects of COVID-19 vaccines before/after vaccination. HCW with low vaccine literacy skills were less likely to recommend COVID-19 vaccines to pregnant women and adults.

Before the COVID-19 pandemic, some studies found that maternal vaccine literacy was related to vaccine hesitancy for children, and that personal vaccine literacy was related to own vaccine acceptance. One study in India showed that maternal health literacy was independently associated with the children’s diphtheria-tetanus-pertussis vaccine status [13]. Moreover, according to a previous Chinese study, parents with higher vaccine literacy were unlikely to be confused by inappropriate information; therefore, they did not tend to be hesitant to accept vaccines [5]. In addition, adult health literacy had a positive connection with high influenza vaccine uptake in the over-65-year-old group [14]. In another study in the USA, people with higher health literacy had higher confidence in the human papillomavirus (HPV) vaccine [15]. Additionally, after the pandemic and beginning of the COVID-19 vaccine programs, high vaccine literacy is associated with higher vaccination of COVID-19 vaccine hesitancy among HCWs in Thailand [4]. Our current study revealed that people with high vaccine literacy had less concerns about low efficacy and severe side effects and had higher intention to recommend vaccines to others. In summary, we found that personal vaccine literacy sometimes affects the vaccine acceptance of not only oneself but also the surrounding people.

It was revealed that there is still room to enhance vaccine literacy of HCWs. In most cases, not all HCWs are familiar with the newly developed COVID-19 vaccines, and they can be sometimes vaccine-hesitant, which eventually leads to vaccine hesitancy of the whole population [2,16,17]. For instance, one study indicated that having the experience of working in medical institutes would not itself raise health literacy [18]. In an Italian study, HCWs, especially nurses and nursing assistants, were vaccine-hesitant despite being healthcare professionals [16]. Regarding the HPV vaccine, which is a relatively new vaccine, even HCWs including doctors were concerned about vaccine safety and efficacy [17]. In our study population, high vaccine acceptance was observed in the higher vaccine literacy group. Therefore, raising the literacy level of HCWs with low vaccine literacy would be effective to enhance the vaccine acceptance of the whole population.

Our study suggested that functional vaccine literacy and interactive-critical vaccine literacy could affect vaccine acceptance differently. To our knowledge, studies regarding the correlation between each type of vaccine literacy and vaccine acceptance are limited. This study found that HCWs with high functional vaccine literacy had lower risk of vaccine-related concerns, and high functional and high interactive-critical vaccine literacy might both contribute to a stronger recommendation of COVID-19 vaccines to others. People with high functional vaccine literacy have higher reading and writing skills, including for comprehension of health information [19]. On the other hand, people with interactive-critical vaccine literacy may communicate and exchange their thoughts with others well, which might lead to their recommendation of vaccines to others who need vaccination. However, in our study, the correlation between high interactive-critical vaccine literacy and reduction in vaccine-related concerns was not significant. This may be because, while high interactive-critical vaccine literacy may lead to right decisions by communicating with people familiar with vaccines, it could also lead to inappropriate decisions to not receive vaccines by communicating only with people skeptical of and opposed to vaccines. This would lead to personal vaccine hesitancy.

Therefore, especially when introducing newer medical technology such as COVID-19 vaccines, government and health agencies should provide appropriate education matched to each health or vaccine literacy level—even to HCWs, who are highly influential among the general population in terms of achieving high vaccine acceptance and high vaccine literacy. Raising vaccine literacy is crucial in the long run; however, raising individual vaccine literacy in a limited short period is assumed to be difficult. Education by providing understandable and accessible information and confirming comprehension levels is thus imperative [12]. Whether people can make the right decisions about their health, including about taking vaccines, is the result of each person’s skill and the demanded knowledge level from healthcare systems [20]. Regarding COVID-19 vaccines, for instance, it is essential for vaccine specialists or infectious disease specialists to provide understandable information to general HCWs.

There are some important points when providing information to HCWs. First, information should not be inclined to specific opinions; that is, education should present both risks and benefits. Second, the amount of information should be appropriate. If this amount is too great, people will be overwhelmed, and if it is too little, people will not be confident of their decision-making ability and will question their decisions [21]. Third, it is practical to train HCWs to educate the general population. HCWs who provide the general population with vaccine information need to acquire communication skills and comprehend each person’s hidden emotions [22]. For instance, only providing knowledge is insufficient to change the minds of vaccine-hesitant people, so adjusting information to each person’s intelligence and background is important [22]. For people originally skeptical of vaccines, communication only with written materials is known to be inefficient; therefore, communication by HCWs to the general population is essential [20]. In fact, in some previous randomized trials, individual education and narrative persuasion both positively contributed to raising vaccine acceptance and reducing vaccine-related fear and concerns [23,24,25].

The present study has some strengths and limitations. First, as a strength, this study revealed that high vaccine literacy would affect HCWs’ intention to recommend that others receive vaccines, including COVID-19 vaccines, as well as their personal vaccine acceptance. At the time of this survey in Japan, COVID-19 vaccines were allocated mainly to HCWs; therefore, HCWs’ influencing power was considered to be strong. Second, our sample size (*n* = 1519) was relatively large, and the response rate to our questionnaire was quite high (91%). Third, the data have been limited on the vaccine literacy of HCWs after the vaccine program began; therefore, our results are valuable.

Our study also has several limitations. First, it was based on a self-administered questionnaire. Therefore, there is some possibility of respondents estimating, and thus declaring, their own vaccine literacy skills incorrectly. Second, this was a cross-sectional survey, so the causalities between vaccine literacy and concerns about vaccines or intention to recommend vaccines to others were not clarified. However, it is highly unlikely that concerns and intentions to recommend vaccines have a reverse effect on low vaccine literacy. Third, this survey was conducted in a pediatric and maternity hospital and a research center. Most HCWs in these settings are engaged in pediatric and maternity medicine; therefore, generalizability to HCWs in other fields and the general population may be low. Because respondents in this study were HCWs focusing on children and pregnant women, their recommendation of vaccines for children and pregnant women likely received more favorable responses as the study population was comfortable with these two groups. Furthermore, the relatively low levels of recommendations for older patients may reflect the respondents’ discomfort in dealing with these groups. Lastly, as the circumstances of the COVID-19 pandemic are changing every moment, our results might be limited.

## 5. Conclusions

Our findings suggest that people with high vaccine literacy have lower risk of having vaccine-related concerns and higher intention to recommend vaccines to others. High vaccine literacy may result in high vaccine uptake. To achieve high vaccine coverage, personalized education is essential, even to HCWs.

## Figures and Tables

**Table 1:** Demographic characteristics of the respondents (*N* = 1519).

Variables		Number (*n*)	Percentage (%)
Gender	Males	337	22.2
	Females	1115	76.0
	N.A.	27	1.8
Age	−30	503	33.1
	31–50	732	48.2
	51-	267	17.6
	N.A.	17	1.2
Occupations	Doctors	391	25.7
	Nurses	639	42.0
	Other paramedics	121	8.0
	Researchers and research assistants	180	11.8
	Office workers and others	171	11.3
	N.A.	17	1.2
K6 scores	Normal: 0–4	1009	66.4
	Mild depressed: 5–8	236	15.5
	Moderate depressed: 9–12	141	9.3
	Severe depressed: 13–24	96	6.3
	N.A.	37	2.5
I got/will get vaccinated against flu.	Before the COVID-19 pandemic (Before 2019/2020 season)	1418	93.3
	Last season (2020/2021 season)	1365	89.8
	This year (2021/2022 season)	1382	90.9
Did you get vaccinated against COVID-19?	Yes	1448	95.3
	No	36	2.4
	N.A.	35	2.4

N.A. = not available.

**Table 2:** Pre-/post-COVID-19 vaccination concerns and intentions to recommend the COVID-19 vaccine to others for each vaccine literacy (functional and interactive-critical) level group.

			Functional Vaccine Literacy Skills			Interactive-Critical Vaccine Literacy Skills		
			High (*n* = 303)	Middle (*n* = 600)	Low (*n* = 582)	High (*n* = 483)	Middle (*n* = 469)	Low (*n* = 509)
	Pre-vaccination concerns							
(1) I may have serious side effects.		*n*(%)	78(25.7)	254(42.3)	263(45.2)	173(35.8)	203(43.3)	212(41.2)
		RR [95%CI]	Ref	1.47 *** [1.22–1.87]	1.50 *** [1.12–1.82]	Ref	1.08 [0.92–1.27]	1.00 [0.85–1.17]
(2) I may have serious subsequent complications.		*n*(%)	22(7.3)	104(17.3)	111(19.1)	67(13.9)	84(17.9)	84(16.5)
		RR [95%CI]	Ref	2.10 ** [1.32–3.34]	2.22 ** [1.40–3.52]	Ref	1.12 [0.82–1.53]	0.99 [0.72–1.36]
(3) Serious side effects may be life-threating.		*n*(%)	39(12.9)	141(23.5)	157(27.0)	107(22.2)	115(24.5)	112(22.0)
		RR [95%CI]	Ref	1.69 ** [1.19–2.38]	1.82 ** [1.29–2.57]	Ref	0.98 [0.77–1.24]	0.81 [0.63–1.03]
	Post-vaccination concerns							
(1) The vaccine may not be effective.		*n*(%)	60(20.7)	152(26.7)	199(36.1)	125(26.8)	140(31.4)	139(29.1)
		RR [95%CI]	Ref	1.22 [0.94–1.59]	1.54 ** [1.12–2.00]	Ref	1.05 [0.85–1.28]	0.94 [0.77–1.16]
(2) Due to vaccines, I may get worse when I get infected.		*n*(%)	3(1.0)	16(2.8)	9(1.6)	6(1.3)	9(2.0)	13(2.7)
		RR [95%CI]	Ref	2.07 [0.63–6.79]	1.04 [0.28–3.78]	Ref	1.74 [0.61–4.94]	2.11 [0.73–6.09]
(3) The vaccines may do some harm in the future.		*n*(%)	56(19.3)	161(28.3)	198(35.6)	114(24.5)	133(29.8)	163(34.1)
		RR [95%CI]	Ref	1.33 * [1.02–1.75]	1.62 *** [1.24–2.12]	Ref	1.10 [0.89–1.37]	1.25 * [1.02–1.54]
(4) I do not worry about anything.		*n*(%)	114(24.5)	133(29.8)	163(34.1)	193(66.6)	304(53.4)	235(42.6)
		RR [95%CI]	Ref	0.86 ** [0.77–0.96]	0.72 *** [0.64–0.82]	Ref	0.91 [0.80–1.02]	0.92 [0.81–1.04]
	Recommend for others							
(1) children (<18 y)		*n*(%)	258(85.2)	502(83.7)	482(82.3)	408(84.5)	390(83.2)	426(83.7)
		RR [95%CI]	Ref	0.98 [0.93–1.04]	0.97 [0.92–1.03]	Ref	0.97 [0.91–1.02]	0.99 [0.94–1.05]
(2) elderly people or people with commodities		*n*(%)	293(96.7)	575(95.6)	547(94.0)	470(97.3)	453(96.6)	470(92.3)
		RR [95%CI]	Ref	1.00 [0.97–1.03]	0.99 [0.92–1.03]	Ref	1.00 [0.97–1.01]	0.97 * [0.94–0.99]
(3) pregnant women		*n*(%)	247(81.5)	450(75.0)	412(70.8)	390(80.8)	351(74.8)	353(69.3)
		RR [95%CI]	Ref	0.94 [0.87–1.00]	0.90 ** [0.83–0.97]	Ref	0.93 [0.87–1.00]	0.88 * [0.82–0.95]
(4) other adults		*n*(%)	297(98.0)	582(97.0)	557(95.7)	468(96.8)	458(97.7)	488(95.9)
		RR [95%CI]	Ref	0.99 [0.97–1.01]	0.98 * [0.96–1.00]	ref	1.01 [0.98–1.03]	0.99 [0.98–1.02]

Tertiles of vaccine literacy: Low group (1–2.25), Middle (2.5–3.25), High (3.25–4). * *p*-value < 0.05, ** *p*-value < 0.01, *** *p*-value < 0.001.

**Table 3:** Continuous analysis for each vaccine literacy (functional and interactive-critical) level group.

	Functional Vaccine Literacy Skills	Interactive-Critical Vaccine Literacy Skills
Pre-vaccination Concerns	RR [95%CI]	
(1) I may have serious side effects.	0.86 ** [0.79–0.94]	1.04 [0.94–1.12]
(2) I may have serious subsequent complications.	0.81 ** [0.69–0.95]	0.99 [0.82–1.21]
(3) Serious side effects may be life-threating.	0.80 ** [0.70–0.91]	1.19 * [1.02–1.39]
Post-vaccination Concerns	RR [95%CI]	
(1) The vaccine may not be effective.	0.81 *** [0.72–0.90]	1.07 [0.94–1.22]
(2) Due to vaccines, I may get worse when I get infected.	1.22 [0.76–1.94]	0.72 [0.42–1.23]
(3) The vaccines may do some harm in the future.	0.82 ** [0.73–0.92]	0.96 [0.84–1.09]
(4) I do not worry about anything.	1.16 *** [1.08–1.24]	0.99 [0.92–1.07]
Intention to recommend to others	RR [95%CI]	
(1) children (< 18 y)	1.02 [0.99–1.05]	1.01 [0.98–1.04]
(2) elderly people or people with commodities	1.00 [0.99–1.03]	1.03 ** [1.01–1.06]
(3) pregnant women	1.05 * [1.01–1.09]	1.08 ** [1.03–1.14]
(4) other adults	1.01 * [1.00–1.03]	1.01 [0.99–1.03]

This table shows relatively RR per 1-point. * *p*-value < 0.05, ** *p*-value < 0.01, *** *p*-value < 0.001.

## Data Availability

The datasets generated during and/or analysed during the current study are not publicly available due to the sensitive nature of data but are available from the corresponding author on reasonable request.

## Supplementary Materials

The following supporting information can be downloaded at: https://www.mdpi.com/article/10.3390/vaccines10091482/s1, Table S1: Questionnaires about vaccine literacy and pre-/post- COVID-19 vaccination worries and intentions to recommend to others for COVID-19 vaccination; Table S2: Principal Component Analysis (PCA) correlations between questions and factors after Varimax rotation (n = 1519).
