# Supplementary material for: Vaccine Literacy, COVID-19 Vaccine-Related Concerns, and Intention to Recommend COVID-19 Vaccines of Healthcare Workers in a Pediatric and Maternity Hospital: A Cross-Sectional Study
Source: Vaccines (Basel). 2022 Sep 6;10(9):1482. doi: 10.3390/vaccines10091482 (PMC9506120; doi:10.3390/vaccines10091482)
Supplement: Supplementary file 1 [file vaccines-10-01482-s001.zip › vaccines-1861277-supplementary.pdf]

Table S1. Questionnaires about vaccine literacy and pre-/post- COVID-19 vaccination worries and intentions to reccomend to others for COVID-19 vaccination.

**Questions about vaccine literacy functional skills**	
When reading or listening to information about current or future COVID-19 vaccines:	
(1) Did you find words you didn't know?<br>(2) Did you find that the texts were difficult to understand?<br>(3) Did you need much time to understand them?<br>(4) Did you or would you need someone to help you understand them?	4-point Likert scale (1-never, 2-sometimes, 3-rarely, 4-often, and in reversed items from 1 often to 4 never)
**Questions about vaccine literacy interactive/critical skills**	
When looking for information about current or future COVID-19 vaccines:	
(1) Have you consulted more than one source of information?<br>(2) Did you find the information you were looking for?<br>(3) Have you had the opportunity to use the information?<br>(4) Did you discuss what you understood about vaccinations with your doctor or other people?<br>(5) Did you consider whether the information collected was about your condition?<br>(6) Have you considered the credibility of the sources?<br>(7) Did you check whether the information was correct?<br>(8) Did you find any useful information to make a decision on whether or not to get vaccinated?	Answers:<br>4-point Likert scale (1-never, 2-sometimes, 3-rarely, 4-often, and in reversed items from 1 often to 4 never)
**Questions about pre-COVID-19 vaccination worries and feelings**	
(1) I may have serious side effects.<br>(2) I may have serious subsequent complications.<br>(3) If I have serious side effects, they may be life-threating.	Answers:<br>4-point Likert scale (Always,Sometimes, Almost Never, Never)
**Questions about whether respondents got COVID-19 vaccines**	
(1) Did you get vaccinated against COVID-19?<br>(2) If you did not get vaccinated, why did not you get vaccinated?<br>(3) If you did get vaccinated, what did you worry about after the vaccinations?	Answers:<br>"Yes" and "No"
**Questions about post-COVID-19 vaccination worries and feelings**	
These question had multiple choices and respondents were able to choose all the choices they agreed with:	
(1) The vaccine may not be effective.<br>(2) Due to vaccines, I may get worse on the contrary when I get infected.<br>(3) The vaccines may do some harm in the future.<br>(4) I do not worry about anything.	Answers:<br>"Yes" and "No"
**Questions about intentions to recommend to others for COVID-19 vaccination**	
(1) children (under 18 years old)<br>(2) elderly people or people with commodities<br>(3) pregnant women<br>(4) other adults	Answers:<br>4-point Likert scale (Always, Sometimes, Almost Never, Never)

Table S2. Principal Component Analysis (PCA) correlations between questions and factors after Varimax rotation (n=1519).

Questions	Factor1	Factor2
1	-0.06	**0.82**
2	-0.05	**0.92**
3	-0.07	**0.90**
4	-0.05	**0.72**
5	**0.67**	-0.02
6	**0.72**	-0.12
7	**0.73**	-0.10
8	**0.67**	-0.09
9	**0.75**	-0.01
10	**0.69**	-0.04
11	**0.67**	-0.07
12	**0.74**	-0.06

Bold fonts mean the greatest correlation. Vaccine literacy functional questions and vaccine literacy interactive-critical questions were shown in Table1 and loaded on two different factors.
